# Prognostic value of serum vascular endothelial growth factor and hematological responses in patients with newly-diagnosed POEMS syndrome

**DOI:** 10.1038/s41408-018-0073-8

**Published:** 2018-04-04

**Authors:** Hao Zhao, Hao Cai, Chen Wang, Xu-fei Huang, Xin-xin Cao, Lu Zhang, Dao-bin Zhou, Jian Li

**Affiliations:** Department of Hematology, Peking Union Medical College Hospital, Chinese Academy of Medical Sciences and Peking Union Medical College, Beijing, China

Polyneuropathy, organomegaly, endocrinopathy, monoclonal gammopathy and skin changes (POEMS) syndrome is a rare plasma cell dyscrasia characterized by high serum levels of vascular endothelial growth factor (VEGF)^1,2^. Bone marrow plasma cells are the likely source of this angiogenic cytokine, which is responsible for the characteristic symptoms of this disorder, including extra-vascular volume overload, hemagiomata and papilledema. A reduction of VEGF level after treatment usually correlates with symptomatic improvement^3–6^. The main treatment strategy is to target plasma cell clones, and monoclonal protein and VEGF levels are used to monitor disease activity.

Hematologic complete response (CR_*H*_) has proved to be a significant predictor of disease outcome. However, some patients without CR_*H*_ still show reasonable survival rates, suggesting that this group is highly heterogenous and requires better prognostic indicators^7^. VEGF response after treatment showed good prognostic value in a retrospective study of 20 patients. Those achieving a normal serum VEGF value after treatment attained prolonged relapse-free survival^8^. However, previous studies were limited by patient number and follow-up time, and lacked OS outcome and comparison with hematologic response.

A total of 476 patients were newly-diagnosed POEMS syndrome and treated at our institute between January 2000 and October 2016. Of these, 190 patients (39.9%, and baseline clinical characteristics were not significantly different from all patients), who had both baseline and post-treatment serum M protein and VEGF data, and whose post-treatment serum samples were collected for half a year since the treatment began, were enrolled in the present study. All patients had been followed for at least 6 months and had elevated baseline serum VEGF levels ( > 600 pg/mL). All patients met the diagnostic criteria proposed by Dispenzieri^2^. Primary therapies included autologous stem cell transplantation (77 patients), melphalan-based therapy (21 patients) and novel agent-based (thalidomide, lenalidomide, bortezomib) therapy (92 patients). The median length of follow-up was 32 months (range, 6–179 months). Detailed clinical features and laboratory information were collected at the time of diagnosis, as described previously^9^. (Online Supplementary Table 1) Serum VEGF was measured with a human Quantikine ELISA Kit (R&D Systems, Minneapolis, MN, USA)^10^. All patients provided informed consent, and the study was approved by the Institutional Review Board of Peking Union Medical College Hospital, in accordance with the Declaration of Helsinki.

The upper limit of the normal serum VEGF range was 600 pg/mL in our institute, as previously described^10^. The complete response of VEGF (CR_*V*_) was a normalization of serum VEGF levels. A VEGF partial response (PR_*V*_) was a reduction of > 50% (baseline must ≥ 1200 pg/mL, 3% patients were between 600 and 1200 pg/mL at baseline). Others were considered as VEGF non-response (NR_*V*_) patients. Hematologic response included CR_*H*_ (a confirmed negative immunofixation electrophoresis (IFE) test and for patients with only light-chain secreting clone also undetectable light chain with serum and urine samples) and no CR_*H*_ (did not meet the criteria for a complete response).

Analyses were performed with SPSS 23 (SPSS Inc., Chicago, IL, USA). The Pearson *χ*^2^ test or Fisher’s exact test were used to ascertain differences between categorical variables. Relationships between baseline factors and VEGF response was compared using a logistic multivariate regression model. Progression-free survival (PFS) and OS were calculated from the start of treatment. Progression was defined as the recurrence or deterioration of clinical symptoms. OS was defined as the time from transplantation to death from any cause. Survival curves were plotted with the Kaplan–Meier method and compared with a log-rank test. Risk factors were analyzed utilizing Cox multivariate models, and the threshold for statistical significance was set at *p* *=* 0.10. Variates which met criteria in univariate analysis, or factors reported prognostic previously were included in multivariate models. Data with *p*-values < 0.05 were considered statistically significant.

There were 17 deaths during follow-up, and other 18 patients had disease progressions. The 3-year PFS was 81.7% and the 3-year OS was 92.8%. Eighty patients (42.1%) achieved CR_*H*_, and the remaining patients (57.9%) had no CR_*H*_. The median time from diagnosis to complete hematologic response was 10 months (range, 1–179 months). If CR_*H*_ was achieved, patients had superior progression-free (*p* = 0.016) and OS rates (*p* = 0.001) compared with patients with no CR_*H*_. (Online Supplementary Figure 1) In CR_*H*_ and non-CR_*H*_ patients, the estimated 3-year PFS was 90.1% and 74.9%, while the 3-year OS were 100.0% and 87.1%, respectively.

The median value of baseline VEGF was 4 764 pg/mL (range, 660–14 328 pg/mL). The mean number of serum VEGF tests carried on any individual patient was 4, and the median interval between each measurement was 7 months. The median time from diagnosis to best VEGF response was 6 months (range, 1–125 months). A total of 112 (58.9%) patients achieved CR_*V*_ after treatment. Fifty-three patients (27.9%) attained PR_*V*_ and the remaining 25 patients (13.2%) had NR_*V*_. According to logistic multivariate analysis, patients with lymphoadenopathy (odds ratio [OR], 0.41; 95% confidence intervals [CI], 0.20–0.84, *p* = 0.014) and IgA type monoclonal protein (OR, 0.45; 95% CI, 0.22–0.93; *p* = 0.032) were less likely to achieve CR_*V*_. These clinical variables, may not be specific but are known to reflect disease burden, and imply that patients with heavy disease are still somehow refractory to modern treatment.

Patients with CR_*V*_ showed better PFS compared with PR_*V*_ patients (*p* = 0.030). However, PR_*V*_ patients had no significant difference compared with NR_*V*_ patients in terms of PFS (*p* = 0.054). NR_*V*_ group included patients with VEGF 600-1200 pg/mL at baseline. The estimated 3-year PFS rates were 87.7%, 79.9%, and 54.8% in CR_*V*_, PR_*V*_ and NR_*V*_ patients, respectively. Patients with CR_*V*_ also had superior OS compared to PR_*V*_ patients (*p* = 0.004), as did PR_*V*_ patients compared with NR_*V*_ patients (*p* = 0.035). The estimated 3-year OS in CR_*V*_, PR_*V*_ and NR_*V*_ patients were 97.4%, 95.1% and 62.8%, respectively. (Online supplementary Figure 2) According to Cox multivariate analysis, the group with CR_*V*_ had superior PFS (HR, 0.38; 95% CI, 0.19–0.77; *p* = 0.008) and OS (HR, 0.12; 95% CI, 0.03–0.44; *p* = 0.001), independent of baseline factors reported previously. (Online supplementary Table 2 & 3)

Next, we compared hematologic and VEGF response by showing the hematologic response distribution in each VEGF response group and *vice versa*. The proportion of hematologic complete response was similar to VEGF response depth, with 62 (55.3%), 17 (32.1%) and 1 (4.0%) patients attaining CR_*H*_ in CR_*V*_, PR_*V*_ and NR_*V*_ groups, respectively. Almost all patients but one case with CR_*H*_ attained CR_*V*_/PR_*V*_. Nearly half of the patients (45.5%) with no CR_*H*_ had CR_*V*_. (Fig. 1) These results suggest that monoclonal plasma cells are still the base of VEGF production but may not be the direct origin. In accordance with this clinical finding, Wang et al. suggested that bone marrow monoclonal plasma cells secret interleukin-6 to promote the proliferation of polyclonal plasma cells and consequent VEGF production in a paracrine circuit^11^. Therefore, targeting plasma cells is the first step in the inhibition of VEGF secretion, however, whether the treatment depth of VEGF inhibition is sufficient for long-term benefit remains unknown.

**Fig. 1:**
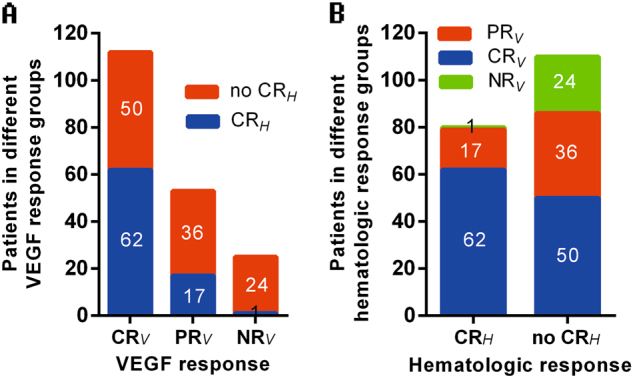
Composition of hematologic and VEGF response groups: (**a**) Hematologic response composition in different VEGF response groups, (**b**) VEGF response composition in different hematologic response groups

We combined PR_*V*_ and NR_*V*_ into a non-CR_*V*_ group to dichotomize VEGF response and combine it with CR_*H*_ to produce a more well-rounded model for prognostic prediction. Patients attained either CR_*H*_ or CR_*V*_ were combined into one group in the Kaplan–Meier survival curve. This group of patients had similar PFS (*p* = 0.932) and OS (*p* = 0.064) compared to patients achieving both CR_*H*_ and CR_*V*_, and showed clearly better progression-free (*p* < 0.001) and OS (*p* = 0.002) than patients with neither CR_*H*_ nor CR_*V*_. (Fig. 2) As mortality is usually caused by organ dysfunction, the normalization of VEGF is supposed to prevent organ damage arising from increased vascular permeability and angiogenesis, which will translate into a long-term benefit. It may be considered that either the complete eradication of plasma cells or the inhibition of VEGF production can derive clinical benefits.

**Fig. 2:**
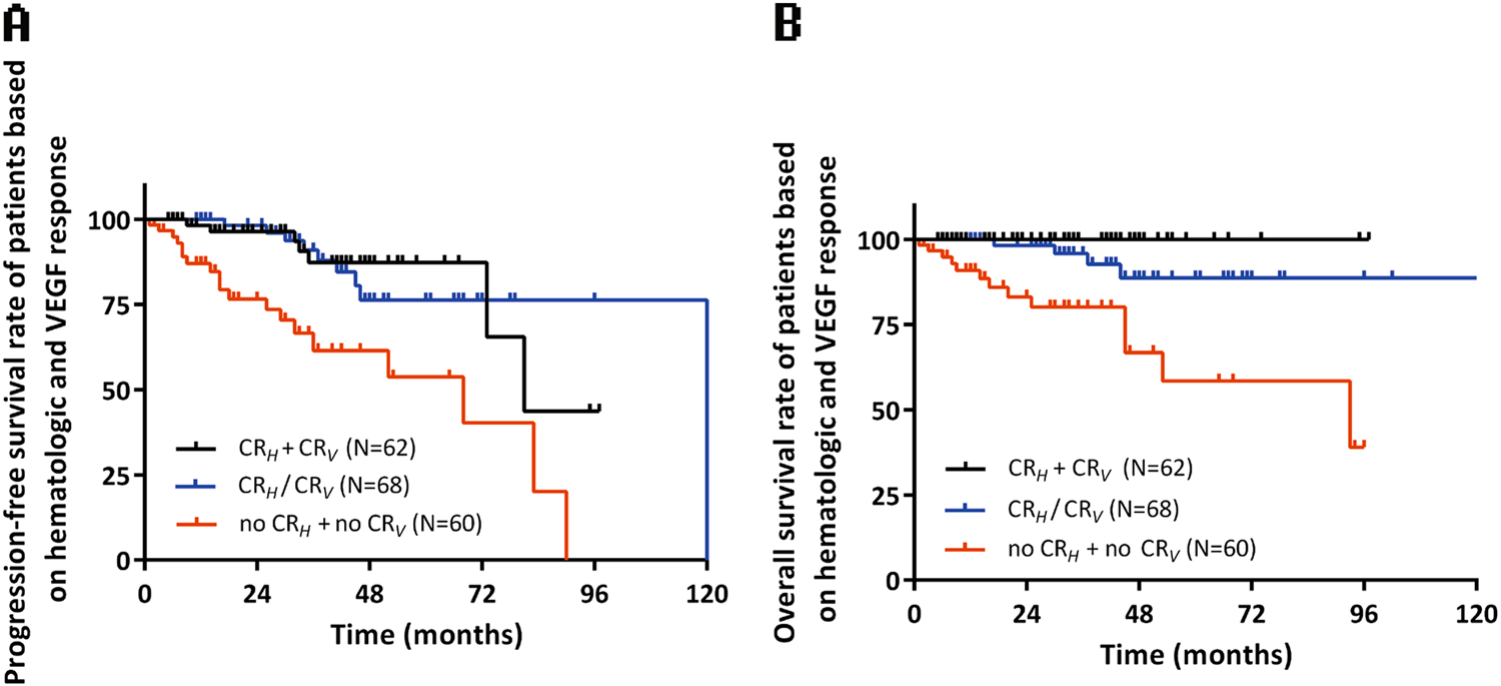
Kaplan–Meier survival curves according to hematologic and VEGF response: (**a**) Progression-free survival curve according to hematologic and VEGF response, (**b**) Overall survival curve according to hematologic and VEGF response

In summary, we demonstrated the prognostic value of VEGF response alone, as well as combined with hematologic response, in POEMS patients, not only re-affirming the importance of regularly monitoring VEGF in routine practice, but also supporting its use as a surrogate endpoint in clinical trials.

## Electronic supplementary material

Supplementary Figure 2

Supplementary Table 1

Supplementary Table 2

Supplementary Table 3

Supplementary Figure 1
