# Supplementary figures and images for: Prognostic value of serum vascular endothelial growth factor and hematological responses in patients with newly-diagnosed POEMS syndrome
Source: Blood Cancer J. 2018 Apr 4;8(4):37. doi: 10.1038/s41408-018-0073-8 (PMC5884844; doi:10.1038/s41408-018-0073-8)

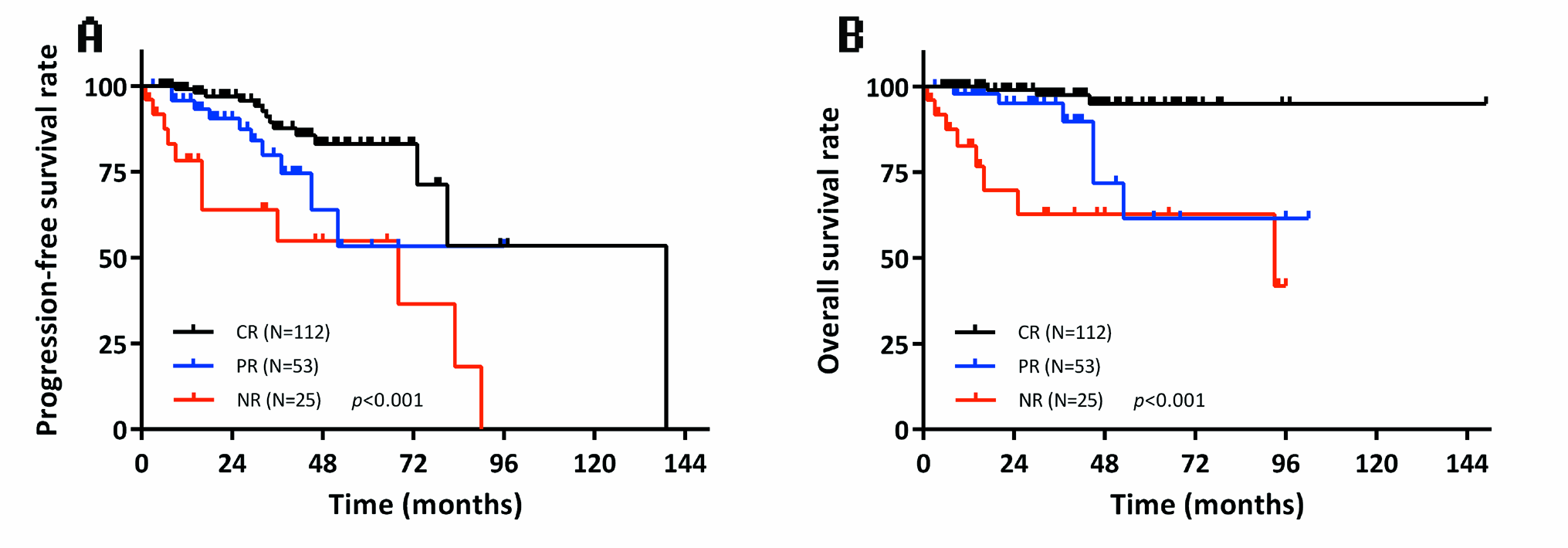

Supplement: Supplementary file 1 — Supplementary Figure 2 [file 41408_2018_73_MOESM1_ESM.tif]

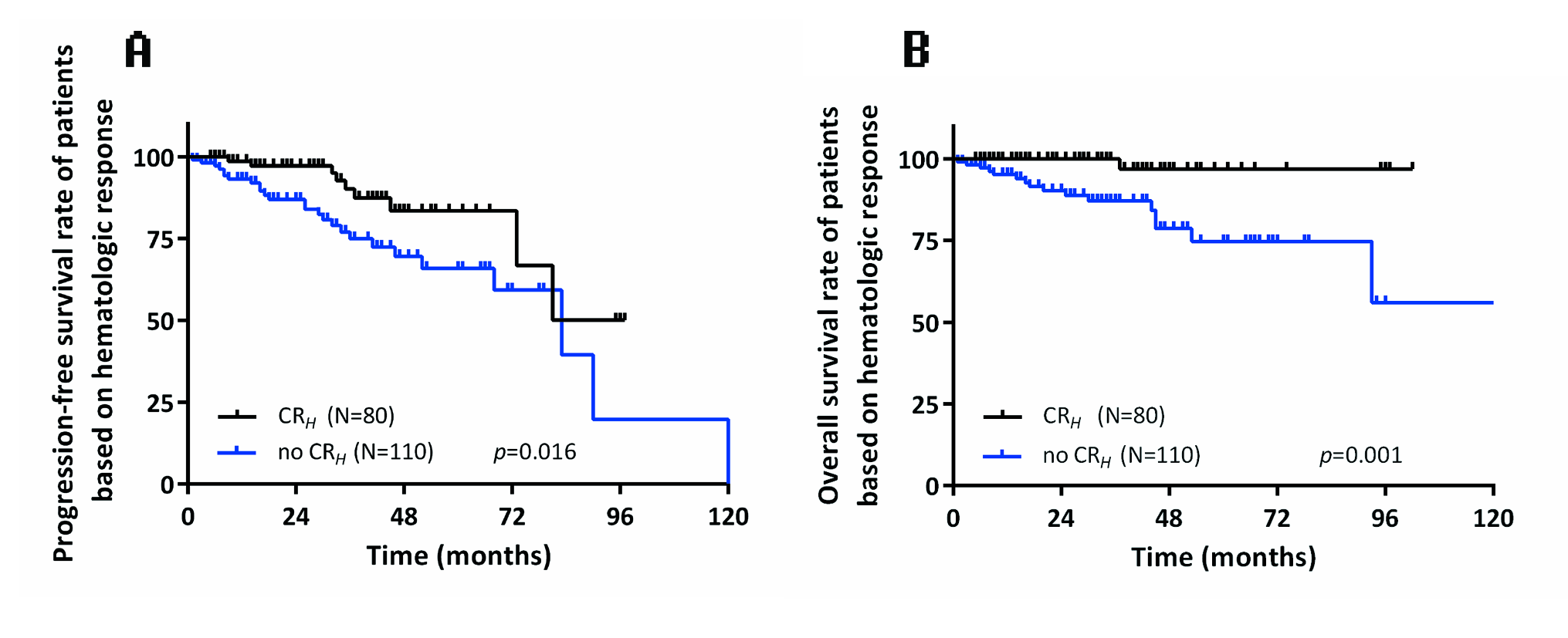

Supplement: Supplementary file 5 — Supplementary Figure 1 [file 41408_2018_73_MOESM5_ESM.tif]
