# Supplementary material for: Prognostic value of serum vascular endothelial growth factor and hematological responses in patients with newly-diagnosed POEMS syndrome
Source: Blood Cancer J. 2018 Apr 4;8(4):37. doi: 10.1038/s41408-018-0073-8 (PMC5884844; doi:10.1038/s41408-018-0073-8)
Supplement: Supplementary file 2 — Supplementary Table 1 [file 41408_2018_73_MOESM2_ESM.docx]

| Prognostic factors | Progression–free survival | |  | Overall survival | |  |
| --- | --- | --- | --- | --- | --- | --- |
|  | HR | CI | *p* | HR | CI | *p* |
| VEGF complete response | 0.32 | 0.159–0.641 | 0.001 | 0.118 | 0.034–0.413 | 0.001 |
| Hematologic complete response |  |  |  |  |  |  |
| Age > 50 years | 0.571 | 0.272–1.198 | 0.691 | 0.763 | 0.282–2.065 | 0.594 |
| pleural effusion | 1.337 | 0.676–2.646 | 0.404 | 1.956 | 0.747–5.118 | 0.172 |
| Pulmonary hypertension | 0.504 | 0.119–2.134 | 0.352 | 1.56 | 0.206–11.780 | 0.842 |
| eGFR< 30 mL/min*1.73 m^2^ | 0.914 | 0.124–6.735 | 0.93 | 2.165 | 0.282–16.596 | 0.457 |
| Alb<30 g/L | 1.374 | 0.474–3.979 | 0.558 | 2.048 | 0.582–7.207 | 0.264 |
| lymphoadenopathy | 0.735 | 0.360–1.500 | 0.397 | 0.752 | 0.283–1.997 | 0.568 |
| IgA type monoclonal protein | 1.054 | 0.501–2.215 | 0.89 | 1.425 | 0.461–4.408 | 0.538 |
| 24 h urine protein>1 g | 1.167 | 0.272–5.017 | 0.835 | 2.953 | 0.666–13.089 | 0.154 |
| ASCT or non–ASCT | 0.897 | 0.432–1.865 | 0.771 | 0.83 | 0.302–2.285 | 0.719 |
