# Supplementary material for: Prognostic value of serum vascular endothelial growth factor and hematological responses in patients with newly-diagnosed POEMS syndrome
Source: Blood Cancer J. 2018 Apr 4;8(4):37. doi: 10.1038/s41408-018-0073-8 (PMC5884844; doi:10.1038/s41408-018-0073-8)
Supplement: Supplementary file 3 — Supplementary Table 2 [file 41408_2018_73_MOESM3_ESM.docx]

| Clinical characteristics | All patients | CR*_V_* patients | no CR*_V_* patients | *p* value |  |
| --- | --- | --- | --- | --- | --- |
|  | (N=190) | (N=78) | (N=112) |  |  |
| Demographic feature |  |  |  |  |  |
| Age > 50 years | 78 (41.1) | 46 (41.1) | 32 (41.0) | 0.995 |  |
| Male | 111 (58.4) | 65 (58.0) | 46 (59.1) | 0.897 |  |
| POEMS features |  |  |  |  |  |
| Polyneuropathy ONLS>4 | 73 (41.0) | 43 (41.0) | 43 (41.1) | 0.985 |  |
| Organomegaly |  |  |  |  |  |
| Hepatomegaly | 82 (43.9) | 56 (51.4) | 26 (33.3) | 0.014 |  |
| Splenomegaly | 116 (62.0) | 63 (57.8) | 53 (67.9) | 0.158 |  |
| Lymphadenopathy | 127 (67.6) | 68 (61.8) | 59 (75.6) | 0.046 |  |
| Endocrinopathy |  |  |  |  |  |
| Diabetes | 24 (13.1) | 17 (15.7) | 7 (9.3) | 0.207 |  |
| Hypothyroidism | 97 (76.4) | 49 (72.1) | 48 (81.4) | 0.219 |  |
| Monoclonal gammopathy |  |  |  |  |  |
| IgA | 124 (65.3) | 64 (57.1) | 60 (76.9) | 0.005 |  |
| SPE >5 g/L ^a^ | 28 (26.7) | 14 (29.2) | 14 (24.6) | 0.595 |  |
| BMPC >10% | 5 (2.7) | 3 (2.7) | 2 (2.6) | 0.658 |  |
| Skin changes |  |  |  |  |  |
| Hyperpigmentation | 171 (91.4) | 98 (89.9) | 73 (93.6) | 0.375 |  |
| Angioma | 115 (61.5) | 66 (60.6) | 49 (62.8) | 0.753 |  |
| Extravascular volume overload |  |  |  |  |  |
| Peripheral edema | 159 (85.9) | 95 (88.8) | 64 (82.1) | 0.193 |  |
| Ascites | 84 (45.7) | 49 (46.2) | 35 (44.9) | 0.855 |  |
| Pleural effusion | 68 (37.2) | 40 (38.1) | 28 (35.9) | 0.761 |  |
| Castleman disease ^b^ | 21 (61.8) | 10 (62.5) | 11 (61.1) | 0.934 |  |
| Papilledema | 90 (67.2) | 53 (66.3) | 37 (68.5) | 0.784 |  |
| Osteosclerosis | 86 (81.1) | 44 (75.9) | 42 (87.5) | 0.127 |  |
| Polycythemia | 4 (2.1) | 4 (3.6) | 0 (0.0) | 0.145 |  |
| Thrombocytosis | 35 (18.4) | 19 (17.0) | 16 (20.5) | 0.535 |  |
| Hypoalbuminemia | 15 (9.5) | 10 (10.3) | 5 (8.2) | 0.659 |  |
| (Alb<30g/L) |  |  |  |  |  |
| Kidney dysfunction |  |  |  |  |  |
| eGFR<30 mL/min/ 1.73m2 | 9 (4.7) | 3 (2.7) | 6 (7.7) | 0.164 |  |
| 24 h urinary protein > 1g | 5 (3.0) | 1 (1.0) | 4 (5.6) | 0.081 |  |
| Initial therapeutic regimen |  |  |  |  |  |
| Melphalan based | 21 (11.1) | 10 (8.9) | 11 (14.1) | 0.263 |  |
| ASCT | 77 (40.5) | 51 (45.5) | 26 (33.3) | 0.092 | |
| Novel agent based | 92 (48.4) | 51 (45.5) | 41 (52.6) | 0.34 |  |
| Abbreviations: ASCT, autologous stem cell transplantation; BMPC, bone marrow plasma cells; Alb, albumin; eGFR, estimated glomerular filtration rate; IgG, immunoglobulin G; ONLS, overall neuropathy limitations scale; FLC, free light chain; SPE, serum protein electrophoresis; VEGF, vascular endothelial growth factor. ^a^ protein exceeding 5 g/L in 28 (26.7%) patients. ^b^ Castleman’s disease was diagnosed in 21 of 34 patients (61.8%) who underwent tissue biopsies. | | | | |  |
