# Supplementary material for: Prognostic value of serum vascular endothelial growth factor and hematological responses in patients with newly-diagnosed POEMS syndrome
Source: Blood Cancer J. 2018 Apr 4;8(4):37. doi: 10.1038/s41408-018-0073-8 (PMC5884844; doi:10.1038/s41408-018-0073-8)
Supplement: Supplementary file 4 — Supplementary Table 3 [file 41408_2018_73_MOESM4_ESM.docx]

| Prognostic factors | Progression | | | Death | | |
| --- | --- | --- | --- | --- | --- | --- |
|  | HR | CI | *P* | HR | CI | *P* |
| VEGF response | 0.261 | 0.113–0.601 | 0.002 | 0.193 | 0.069–0.545 | 0.002 |
| eGFR< 30 mL/min*1.73 m^2^ | 0.577 | 0.073–4.546 | 0.602 | 1.454 | 0.178–11.857 | 0.727 |
| Age > 50 years | 0.475 | 0.193–1.172 | 0.106 | 0.898 | 0.328–2.461 | 0.835 |
| Pleural effusion | 1.37 | 0.597–3.148 | 0.458 | 1.342 | 0.452–3.987 | 0.596 |
| Pulmonary hypertension | 0.291 | 0.060–1.415 | 0.126 | 0.393 | 0.048–3.246 | 0.386 |
| Albumin < 30 g/L | 0.833 | 0.226–3.065 | 0.783 | 1.667 | 0.394–7.053 | 0.488 |
| ASCT | 0.796 | 0.337–1.883 | 0.604 | 0.5667 | 0.181–1.763 | 0.326 |
